# What goes up must come down: EEG phase modulates auditory perception in both directions

**DOI:** 10.3389/fpsyg.2013.00016

**Published:** 2013-01-28

**Authors:** Rufin VanRullen, Douglas McLelland

**Affiliations:** ^1^Centre de Recherche Cerveau et Cognition, Université Paul Sabatier, Université de ToulouseToulouse, France; ^2^CNRS, CerCoToulouse, France

**A commentary on**

**A precluding but not ensuring role of entrained low-frequency oscillations for auditory perception**

by Ng, B. S., Schroeder, T., and Kayser, C. (2012). J. Neurosci. 32, 12268–12276.

Recent years have seen mounting evidence that the phase of low-frequency EEG oscillations—particularly in the theta (4–8 Hz) and alpha (8–13 Hz) frequency ranges—is closely related to visual perception (Busch et al., 2009; Mathewson et al., 2009; Busch and VanRullen, 2010; Drewes and VanRullen, 2011; Dugue et al., 2011; VanRullen et al., 2011; Chakravarthi and VanRullen, 2012; Hamm et al., 2012; Romei et al., 2012; Schyns et al., 2012). In a recently published article, Ng and colleagues report, for the first time, that auditory perception also depends on the phase of theta EEG oscillations (Ng et al., 2012)—an exciting novel finding suggesting that brain oscillations have perceptual consequences in multiple sensory modalities. This successful demonstration after previously failed attempts by other groups (Zoefel and Heil, unpublished) (Ilhan and VanRullen, unpublished) may be owed, in part, to the analysis of EEG oscillations that were not spontaneously produced by the brain, but rather evoked (or “entrained”) by an auditory background stimulation (Large and Jones, 1999; Lakatos et al., 2008; Henry and Herrmann, 2012). In line with this idea, another recent report indicated that entrainment of brain oscillations by 10 Hz periodic transcranial electric stimulation of auditory regions induces a periodic fluctuation of auditory sensitivity at the same frequency (Neuling et al., 2012).

While the primary finding of a periodic modulation of auditory perception by EEG oscillations is a major novel result that we would not wish to contest, there is another conclusion of the study by Ng et al. (2012) that merits elaborating: the EEG phase modulation they reported was higher for misses (trials in which the auditory target was present but undetected) than for hits (detected targets). Based on these unexpected results they proposed a “precluding” model of phase modulation, in which the “duty cycle” of phase modulation would be significantly longer than 50% (we define “duty cycle” as the proportion of a full cycle for which performance is enhanced compared to baseline; a sinusoidal modulation would result in a 50% duty cycle). In their model, the short period of the cycle during which performance is impaired (less than 50% of the cycle) would create a “window of no-opportunity” for auditory perception, while the remaining phases (the majority of the cycle) would all be equally favorable to perception. Based on this “precluding” model the authors were able to account for the more intense modulation observed for misses.

**Figure 1:**
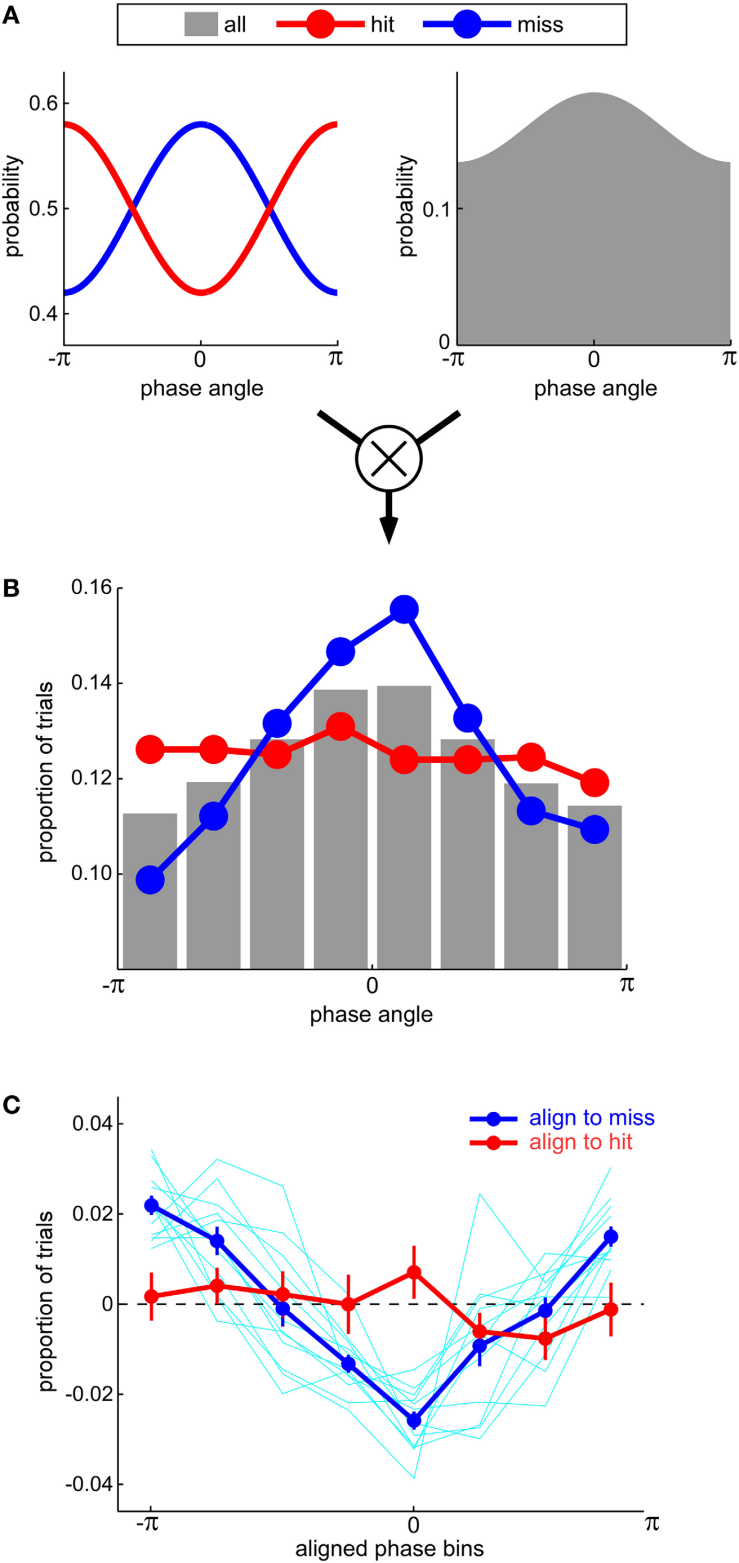
**(A)** An equal modulation of hit and miss rates by oscillatory phase (left) can result in an apparent imbalance between hits and misses if it is accompanied by a non-uniform prior probability distribution of phase values sampled across all trials (right). This non-uniform distribution may arise from a phase-reset of oscillations by the experimental stimulation and/or an inadequate sampling of auditory performance. **(B)** Because the most likely phase value also happens to correspond to the angle that minimizes hit rate (and maximizes miss rate), the number of hits across phases (red curve) is near-uniform; in contrast, the number of misses (blue curve) is strongly biased toward this expected phase. The data in this panel correspond to one simulated subject in the experiment by Ng et al. (2012). The simulation directly relies on the phase modulation and the prior distribution of phases depicted in panel **(A)**. **(C)** As in the study by Ng et al. (2012), the difference between the distributions of hits and misses for 12 simulated subjects were averaged after each subject's phase angles were aligned to the phase that maximized misses (blue curve) or hits (red curve). Error bars denote s.e.m. across subjects. A clear modulation is only apparent when phases are aligned to the angle that maximizes misses, not hits.

There is an inherent conceptual contradiction, however, in proposing that misses can be more modulated by oscillatory phase than hits. Since any target-present trial is either a hit or a miss, any increase in the probability of a miss at certain phases should normally be accompanied by an equivalent decrease in the probability of a hit, and vice-versa. The probabilities of hits and misses should thus be modulated by oscillatory phase by the same amount, only in opposite directions. How could Ng et al. (2012) observe a larger effect for misses than for hits? We suggest that this happened because these authors based their calculations on the relative number of hits and misses observed around each phase value, without taking into account the fact that all phase values were not equally sampled in their paradigm (Figure 1). In mathematical terms, they measured *p(miss*&*phase)*, the joint probability of observing negative performance at a given phase, but drew conclusions about *p(miss*|*phase)*, the actual probability of observing negative performance given a certain phase; as according to Bayes' law, *p(miss*|*phase)* = *p(miss*&*phase)/p(phase)*, it appears these authors neglected to take into account the prior distribution of phases across trials, *p(phase)*. If we postulate that the non-uniform prior distribution of phases was, perhaps by chance, biased toward a phase value that was detrimental for auditory perception (Figure 1A; see also Figure 3D in Ng et al., 2012), then the alignment of the prior distribution peak with the peak of the miss probability will result in an enhanced phase modulation of the joint probability distribution for misses; in contrast, the alignment of the prior distribution peak with the trough of the hit probability will suppress the phase modulation of the joint probability distribution for hits. In this way, the results of Ng et al. (2012) can be accounted for (Figures 1B and C) without resorting to a “precluding” model with an unconventional duty cycle (indeed, our simulations relied on a standard sinusoidal modulation with a 50% duty cycle). We readily acknowledge, naturally, that this alternative account does not categorically rule out a possible imbalance in the duty cycle—only future direct experimental measurements of this duty cycle can resolve this question.

Why was the phase non-uniformly distributed across trials? The clever paradigm used by Ng et al. (2012) should in theory guarantee such a uniform distribution. They presented their auditory target at one of six different moments following the onset of an auditory noise sequence (1500–2500 ms in steps of 200 ms). With the assumption that the sequence onset would reset a theta oscillation at 4 Hz, these chosen moments should have provided a uniform sampling of 4 Hz oscillatory phases. Unfortunately, this ingenious paradigm critically depends on the assumption that the relevant oscillation—the one that is phase-reset by stimulus onset and subsequently modulates auditory perception—is precisely at 4 Hz. The corresponding oscillatory band was extracted from EEG data filtered between 2 and 6 Hz, implying that the relevant oscillation might lie anywhere within this range. Any departure from the assumed value of 4 Hz would unfortunately have produced a non-uniform phase distribution (for example, with a relevant oscillation at 5 Hz, which was also the rate of sampling of auditory performance in this paradigm, only a single phase value would be recorded across the different samples).

If our account is correct, then the asymmetric effect of phase on auditory perception (stronger for misses than for hits) is a direct consequence of the unfortunately biased sampling of auditory performance around a phase value that impairs performance. We thus predict that this asymmetry will be reversed if the experiment is repeated but sampling auditory perception at six delays between 1600 and 2600 ms (by steps of 200 ms) instead of between 1500 and 2500 ms. At these delays, the non-uniform sampling of phases will be biased toward a phase that favors hits rather than misses. This prediction should be properly tested before embracing the less parsimonious “precluding” model of phase modulation. In the meantime, we renew our congratulations to Ng and colleagues for demonstrating that brain oscillations have perceptual consequences not just in the visual, but also in the auditory domain.
